# The Epidemiological, Morphological, and Clinical Aspects of the Cervical Ribs in Humans

**DOI:** 10.1155/2016/8034613

**Published:** 2016-11-15

**Authors:** Łukasz Spadliński, Tomasz Cecot, Agata Majos, Ludomir Stefańczyk, Wioletta Pietruszewska, Grzegorz Wysiadecki, Mirosław Topol, Michał Polguj

**Affiliations:** ^1^Department of Angiology, Interfaculty Chair of Anatomy and Histology, Medical University of Łódź, Narutowicza 60, 90-136 Łódź, Poland; ^2^Centre for Learning Anatomical Sciences, Faculty of Medicine, University of Southampton, University Road, Southampton SO17 1BJ, UK; ^3^Department of Radiological and Isotopic Diagnosis and Therapy, Medical University of Łódź, Ul. Żeromskiego, 11390-549 Łódź, Poland; ^4^Department of Radiology, Medical University of Łódź, Kopcińskiego 22, 90-153 Łódź, Poland; ^5^Department of Otolaryngology and Oncological Laryngology, Medical University of Łódź, Kopcińskiego 22, 90-153 Łódź, Poland; ^6^Department of Normal and Clinical Anatomy, Interfaculty Chair of Anatomy and Histology, Medical University of Łódź, Narutowicza 60, 90-136 Łódź, Poland

## Abstract

A familiarity with the anatomy of some types of bone anomalies is necessary for clinicians involved in many medical areas. The aim of this paper is to review the newest literature concerning the morphology, embryology, clinical image, and therapeutic methods of the cervical ribs in the humans. The incidence of cervical ribs has been found to vary from 0.58% in Malaysian population to 6.2% in Turkish population. Cervical ribs have clinical implications that are generally divided into neurological or vascular. This study is of particular importance for clinicians, as early identification of cervical ribs may prevent life-threatening complications.

## 1. Introduction

**Figure 1:**
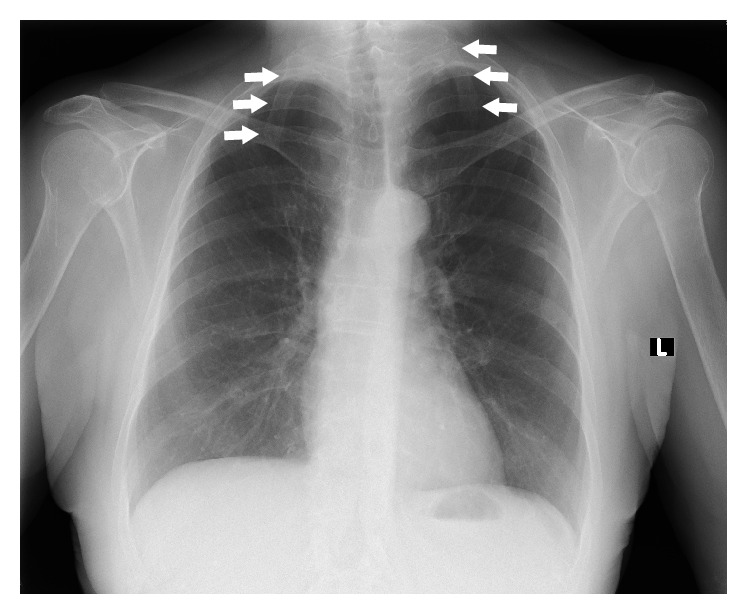
Chest radiograph showing bilateral cervical ribs (arrows) attached to the seventh cervical vertebra.

**Figure 2 fig2:**
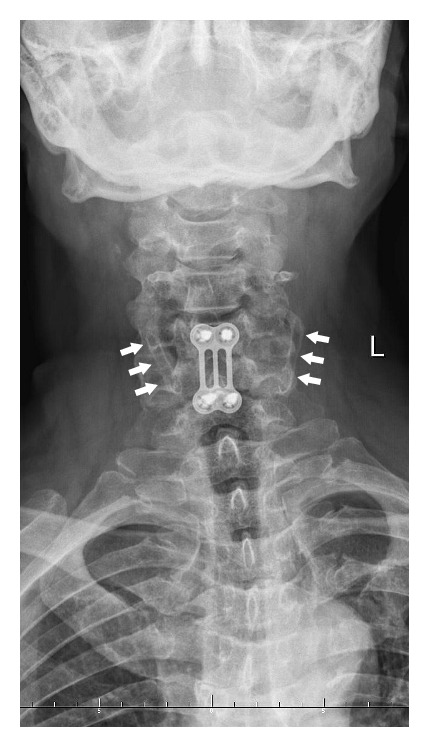
Radiograph of the cervical column showing bilateral cervical ribs (arrows) attached to the fourth cervical vertebra. Patient after neurosurgical intervention in cervical spine.

The cervical rib is described as an anomalous, supernumerary, extra, or additional rib which arises from the seventh cervical vertebra (Figure 1). However, according to Tubbs et al., a cervical rib could also originate from the sixth or the fifth cervical vertebrae [1]. In our practice, we have even found that, in very rare instances, it can also originate from the fourth cervical vertebra (Figure 2). Cervical rib is also known as “Eve's rib” [2]. Although cervical ribs are known to commonly occur in reptiles [3], they are rare in humans. On the other hand, cervical ribs in comparison to very rare skeletal variations like double suprascapular foramen are common anomaly [4]. Radiograph images indicate that the prevalence of cervical ribs is below 1% in the general population; however, studies have found its occurrence to vary significantly from 0.58% to 6.2% depending on the population [5, 6]. Surveys indicate that cervical ribs commonly occur bilaterally and are more frequent in women [7]. However, it is also reported that cervical ribs are typically unilateral, most commonly occurring on the right side [8], and if the cervical ribs are bilateral, they are often asymmetrical (Figure 1).

In approximately 50% of the patients with a complete cervical rib, clinical manifestations begin spontaneously [9]. The presence of cervical ribs may be associated with diminished or absent radial pulse (especially when the arm is abducted), reduced sensation, or a painful and weakened hand. Patients may also complain of tingling and numbness of the ulnar part of the forearm and hand. Cervical ribs may mimic palpable mass in the supraclavicular fossa or may be presented as pulsation due to displacement of the subclavian artery [9–11].

The aim of the study was to review the morphology and embryonic development of cervical ribs, as well as their clinical implication, diagnostic process, and therapeutic methods.

## 2. Embryonic Development of Cervical Ribs

The skeletal system arises from the paraxial mesoderm. The cells of the mesoderm give rise to somites on either side of the neural tube. Sometimes, they divide into a ventral part, the sclerotome, and a dorsal part, the dermatome. At the end of the fourth week of embryogenesis, the cells of the sclerotome convert into mesenchymal cells and then into ribs.* Hox* genes are responsible for patterning of the axial skeleton, and mutations within them probably are implicated in the development of cervical ribs [12]. It has been hypothesized that abnormality in the expression of* Hox* genes could influence oncogenesis. Germ cell tumors, astrocytoma, and acute lymphoblastic leukemia were diagnosed in children with higher rate of such cervical anomalies as cervical ribs. A greater difference occurs in the frequency of cervical anomalies (transverse apophysomegaly and cervical ribs): 8.6% versus 6.1%, respectively (*p* = 0.047) [13].

The cooccurrence of absent or rudimentary 12th ribs in 23.6% of the cases with cervical ribs indicates that, in approximately 8% of the fetuses, a homeotic shift occurred over a larger part of the vertebral column. Such observation suggests that the expression of multiple Hox genes may have been affected these fetuses [14].

One of the significant genes that control skeletal formation is growth differentiation factor 11 (GDF11) [15]. Knockout of GDF11 function causes abnormal patterning of the anterior/posterior axial skeleton. Li et al. [15] demonstrated significant effects of GDF11 propeptide transgene on vertebral formation, which are likely occurring through depressing GDF11 function and altered locations of Hoxa-4 and Hoxa-5 expression. Takihara et al. [16] indicated that the rae28 gene is involved in the regulation of Hox gene expression and segment specification during paraxial mesoderm and neural crest development.

Normal cervical vertebrae in mammals, including humans, have pleurapophyses, small parts that are homologous to the cervical ribs in the ancestors of mammals and these tiny rudimentary rib parts are fused with the remaining part of the transverse process [17]. The normally tiny cervical rib part forms as a larger rib, with a highly variable phenotype often asymmetric between the left and right side. There is the tendency of the cervical rib to fuse and as it is normally too small to reach the sternum, it fuses sometimes with the first rib but also often with the transverse process of C7 itself (apophysomegaly) [17].

Ten Broek et al. [18] suggested that locally perceived developmental signals are more important for the developmental outcome than the origin of the cells. Scientists found a strong coupling between the abnormality of the vertebral patterns and the amount and strength of associated malformations; that is, the longer the disturbance of the vertebral patterning has lasted, the more associated the malformations have developed and the more the organ systems are affected. According to Kjær and Hansen [19], the presence of unilateral or bilateral cervical ribs was a constant finding which seems applicable as a phenotypic characteristic of Ullrich-Turner syndrome.

## 3. Detection of Cervical Ribs

The presence of cervical ribs was first observed by Galen in the second century, during dissections of human cadavers [20]. However, the first observations of the clinical manifestations of neurovascular compression caused by cervical ribs were made by Cooper in 1818 [21].

**Figure 3 fig3:**
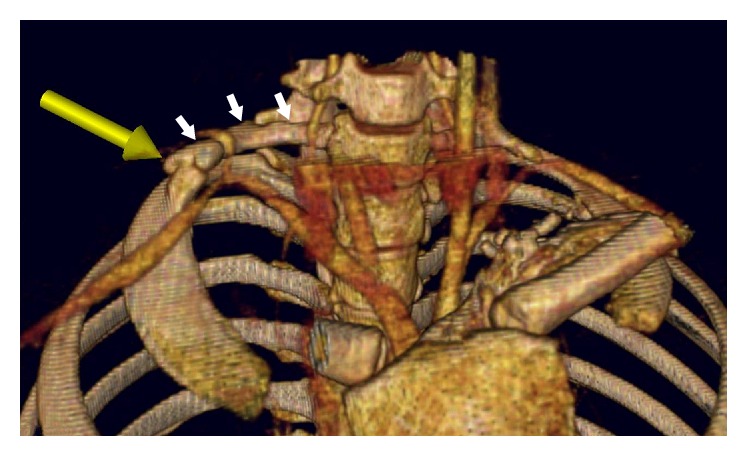
Three-dimensional volume rendering (VR) chest multidetector computed tomography (MDCT) demonstrating unilateral cervical rib (white arrows) and pseudoarthrosis (yellow arrow). The course of the subclavian artery is changed by unusual topographic relations.

**Figure 4 fig4:**
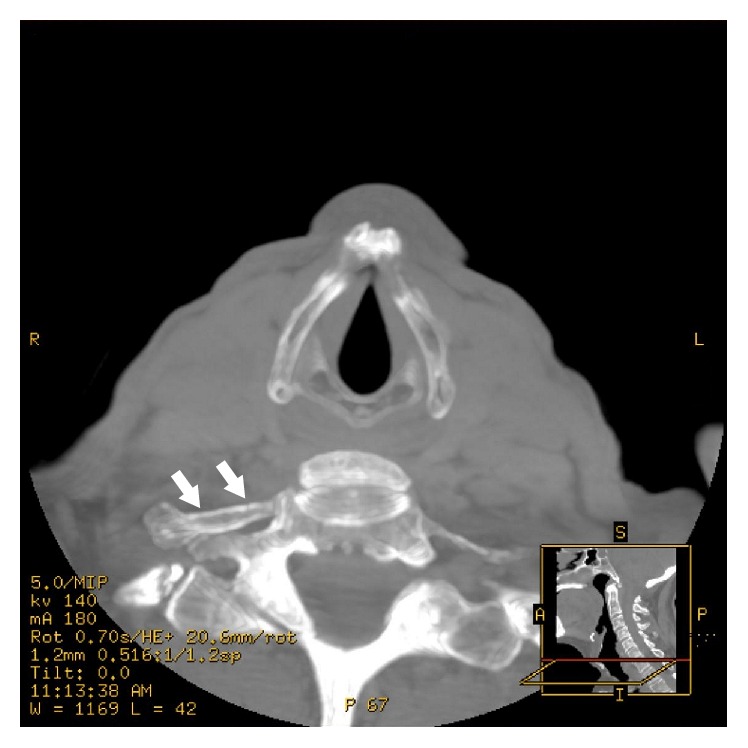
A computed tomography transverse (CT) scan at the level of the seventh cervical vertebra demonstrating unilateral cervical rib (arrows).

**Figure 5:**
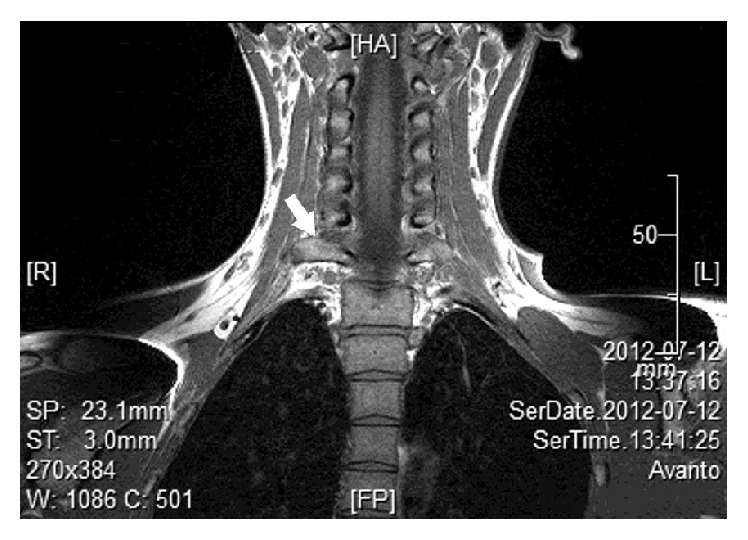
A magnetic resonance imaging (MRI) coronal scan demonstrating unilateral cervical rib (arrow).

Most commonly, cervical ribs are detected in X-ray films of the chest or the cervical part of the vertebral column (Figures 1 and 2). According to Viertel et al., 74.5% of cervical ribs are overlooked in cervical spine computed tomography (CT) examinations [22]. Nevertheless, three-dimensional computed tomography is believed to be invaluable for identifying the cervical ribs. It significantly improves the evaluation of their attachment and length [23] and it could be considered the “gold standard” for cervical rib detection (Figure 3). However, cervical rib is also good visible on “classical” computed tomography transverse scans (Figure 4). Alternatively, magnetic resonance imaging due to its excellent spatial resolution allows the detection of the fibrous band which may connect the distal end of cervical rib with the first thoracic rib [24]. Cervical ribs are usually detected in the middle-aged adult [25]. Sometimes, different methods of diagnosis including X-ray, MRI, or CT imaging are essential to prevent skipping of diagnosis (Figures 1 [Fig fig2] [Fig fig3] [Fig fig4]–5).

Demonstration of the fetal ribs by two-dimensional (2D) sonography is not simple. However, increasingly in the last decade, three-dimensional (3D) ultrasound has been used as part of the fetal organ examination [26, 27]. Hershkovitz [27] recommended using 3D ultrasound with maximal mode rendering for detection of the cervical ribs because it is not time-consuming and is very simple.

## 4. Morphological and Demographic Aspects of Cervical Ribs

The cervical rib consists of a head, neck, and tubercle, with the shaft sometimes being present. It attaches posteriorly to the first rib, near to the insertion of the anterior scalene muscle, and is typically connected by a fibrous band [24]. This fibrous band, if not calcified, is invisible on radiograms. In some cases, the hypertrophied scalene tubercle of the first rib could participate in articulation with the cervical rib [28]. Cervical ribs could also be attached to the first rib by pseudoarthrosis, which is a false joint. Such junctions are observed to be completely developed in only 25% of those with cervical ribs [28]. Unilateral cervical ribs were found more frequently on the left side than on the right side [14].

There are two types of cervical ribs: those which are complete and articulate with the first rib and those which are incomplete and end freely in the soft tissues of the neck. Cervical ribs must articulate with the transverse process; if the articulation does not occur, the structure is identified as an elongated transverse process or transverse apophysomegaly [29].

The prevalence of cervical ribs depends on the population. Previous studies have found the occurrence to range from 0.58% in Malawian population [5] to 6.2% in Turkish population [6] (Table 1). The situation is complex when considering a population like that of the UK or USA, with high proportions of immigrants. In 2009, Brewin et al. assessed 1,352 chest radiographs of London residents of various genders and ethnicity [30]. They noted that the prevalence of cervical ribs was 0.74% throughout the entire population. A higher prevalence was observed in female than male residents (1.09% and 0.42%). A similar study performed on an American population found the occurrence of cervical ribs to be 0.5–1% [3].

In Anatolian population (Turkey), the prevalence of the cervical rib was found to be 3% [36]. Higher occurrence was noted in women (2.17%) than men (0.83%). Incomplete cervical ribs were found in 2.24% of cases, which was three times higher than the incidence of complete ones (0.76%). Cervical rib predominated in female participants, with a prevalence of 2.17%. Bilateral cervical rib (1.49%) was as frequent as unilateral (1.51%). In unilateral cases, the right-sided rib dominates [36]. An even higher prevalence of cervical ribs was noted in a survey by Erken et al. in Turkish population, with an occurrence of 6.2% [6].

In Saudi Arabian population, the occurrence of cervical rib was found to be 3.4% with a female predominance (2.01 : 1). Bilateral cervical ribs were found in 1.4% of cases, and unilateral cervical ribs were observed more frequently on the right side (1.1%) [37]. In Nigerian population, the prevalence of cervical ribs was lower (0.65%), with a slightly higher occurrence in female (0.36%) than male subjects (0.29%). Unilateral ribs were found more often (0.43%), with the right side dominating (0.29%) [32]. These results concur with those of Abimbola and Willido [8] which indicate that cervical ribs were present in 0.6% of patients from Urhobo population (Nigeria). They also indicate a higher occurrence in females than males (F: 0.78%; M: 0.4%, resp.) [8]. Similarly, the occurrence of cervical ribs in Malawian population was found to be 0.58%, with unilateral and bilateral cervical ribs occurring equally frequently (0.29%). Of the unilateral ribs, the left side was more frequent (0.2%). Female subjects were more likely than male subjects to possess cervical ribs (0.39%) [5].

One of the largest studies on the prevalence of cervical ribs undertaken so far was performed in 2013 by Sharma et al. on 5000 chest radiograms of residents from India [25]. In total, 61 persons were found to possess cervical ribs, which constitutes 1.22% of the study population. Cervical ribs were more commonly unilateral (0.78%) than bilateral (0.44%). Although the majority of surveys report that cervical ribs predominate in women, Sharma et al. found them to be more common in males (M: 0.68%; F: 0.54%). In Sharma et al.'s study, no significant correlation was found between the prevalence of cervical ribs and sex or body side in a population from central India [25]. However, another survey conducted in an Indian population around Lucknow by Gupta et al. revealed the prevalence of cervical ribs to be half of that of the previous mentioned study (0.6%) [31]. A higher frequency was observed in female (0.73%) than male subjects (0.49%). Bilateral cases were more frequent. Bilateral cervical ribs were found in 2 of 77 (2.59%) adult cadavers in another Indian study [34]. Finally, in a study on Kashmiri population, the prevalence of cervical ribs was found to be 2.67%. Unilateral cervical ribs (1.76%) were more common than bilateral ones (0.91%), with the right side dominating. Cervical ribs were found to be more common in women (1.55%) than men (1.12%) [35]. Those four surveys illustrate the significant variation in the prevalence of cervical ribs in the population of India (Table 1).

The prevalence of cervical ribs in a population in Chennai (India) was found to be 1.16%, with unilateral occurrence in 20 of 22 cases. However, in contrast to most surveys, the prevalence was found to be higher in men than women, with 19 of the 22 subjects being male and most cases being asymptomatic [33].

Study of healthy Caucasian children found the prevalence of cervical ribs to be 2.2% [13]. A three-dimensional ultrasound study revealed the occurrence of cervical ribs to be 1.4% in fetuses [27].

## 5. Clinical Implication of Cervical Ribs

According to Sandring [38], excessive growth of the anterior tubercle of the seventh cervical vertebra is responsible for the development of cervical ribs. It has been reported that all fetuses have cervical ribs, which disappear before childbirth [39]. Furtado et al. report a higher prevalence of cervical ribs (43.1%) in stillborn fetuses compared with live-born ones who died in the first year (11.8%) and suggest that cervical ribs may be markers of disadvantageous developmental events occurring during critical and conserved stages of blastogenesis that are implicated in both cervical segmentation defects and fetal mortality [40]. Galis et al. report that the mortality rate before birth of fetuses and neonates with a cervical rib is 78% [41]. A survey of 199 electively aborted fetuses in Finnish population (10–21 hbd) showed that 37.7% had cervical ribs, with bilateral predominance [14].

Homeotic transformations that change the number of cervical vertebrae are extremely common in humans but are strongly selected because almost all individuals die before reproduction. Selection is most probably indirect, caused by a strong coupling of such changes with major congenital abnormalities [29, 41].

Schumacher et al. [42] speculated that minor aberrations might also manifest in other morphological defects, especially in minor anomalies of the ribs. Scientists reviewed chest roentgenographs of 1000 children with malignancies for rib anomalies and compared them to 200 patients with mainly infectious diseases. They found 242 rib anomalies in 218 children with tumors (21.8%) and 11 (5.5%) in children without malignancy. A high incidence of cervical ribs was found in neuroblastoma (33%), brain tumor (27.4%), leukemia (26.8%), soft tissue sarcoma (24.5%), Wilms tumor (23.5%), and Ewing sarcoma (17.1%) [42].

The presence of cervical ribs is usually asymptomatic (90% of patients) and does not require their removal. Factors like trauma, overuse, poor posture, or the presence of large breasts predispose to symptoms [43]. If symptomatic, cervical ribs have numerous clinical implications, although they can be generally regarded as either neurological or vascular. According to Kataria et al., most patients with cervical ribs suffer due to compression of the brachial plexus [44]. Another survey conducted by Dashti and Ghasemi also indicates that neurological manifestations were more frequent than vascular [10]. The type of manifestation depends on the morphology of the cervical ribs. It is commonly known that incomplete ribs only affect the brachial plexus, whereas complete ribs also have an impact on the subclavian artery.

Patients with a neurological manifestation may complain of intermittent, migrating pain in the affected upper limb and paresthesia and numbness in the affected ulnar side of the forearm. A cervical rib may distort the lower part of the brachial plexus, especially the eighth cervical and first thoracic nerve roots. Cervical ribs may compress the nerves of the brachial plexus and slightly weaken motor strength of the muscles of the forearm and hand. Compression of the nerves may be especially manifested at the forearm, thenar, hypothenar, and interosseus muscles, or clawing of the little, ring, and middle fingers. Sometimes patients may present with difficulty in holding a pen. Moreover, it has been noted that cervical ribs may also induce subclinical nerve damage [45]. A correct diagnosis of compression of the brachial plexus elicited by the cervical rib should include the exclusion of carpal tunnel syndrome, neuritis of ulnar nerve, or entrapment syndromes of the prolapsed ulnar or cervical disc [8].

Patients with vascular manifestation may complain of mild discoloration of the hand, claudication, or dizziness. Furthermore, the cervical ribs may induce diminished distal pulses at wrist, prolongation of capillary refill, and even gangrenous changes at the finger tips. Symptoms of ischemia may be diminished by the formation of collaterals, which makes diagnosis difficult. Systolic blood pressure may be decreased and bruits may be heard. In the case of compression of the subclavian artery by cervical rib, the Adson test may be positive during hyperabduction of the limb. In young athletes, exercises with hyperabduction can often elicit symptoms of compression adjacent structures by cervical rib [3].

The presence of a cervical rib described in case reports may result in distal and cerebral embolism, recurrent stroke in the young, cerebellar infarction, subclavian aneurysm (very rare), subclavian artery thrombosis, upper extremity venous thrombosis, secondary Raynaud's phenomenon, and ischemia of the upper limb with gangrene of the distal phalanx [44].

The treatment of conditions associated with the presence of cervical ribs depends on the symptoms. Conservative treatment relies on alleviation of the pain by dosing analgesics (NSAIDs), the use of muscle relaxant drugs, physiotherapy, and making changes to lifestyle. The aim of physical therapy is to strengthen the muscle of the shoulder girdle [45]. Conservative treatment is recommended as initial treatment, unless acute vascular or neurological manifestations are observed. However, if conventional treatment does not alleviate the symptoms, surgical treatment is required. The first resection of a cervical rib was performed in 1861 [46]. Nowadays, surgical procedures on the cervical rib can be performed using the supraclavicular or the transaxillary approach. Surgical treatment by supraclavicular excision is preferred due to safety and good exposure of cervical rib [47]. Anterior scalenotomy is often performed to enable easier access to the subclavian artery. However, in some cases, it is also necessary to remove the first rib.

## 6. Conclusion

The occurrence of cervical ribs varies broadly and depends on ethnicity. However, the prevalence of cervical ribs is similar within an individual population. It is important to consider the presence of a cervical rib in all medical specializations, as its early identification may prevent life-threatening complications.

## Figures and Tables

**Table 1:** The prevalence of cervical ribs according to population. CR: cervical rib.

Prevalence of CR [%]	Population	Author
0.58%	Malawian	Ebite et al. [5]
0.6%	Nigerian (Urhobo)	Abimbola and Willido [8]
0.6%	Indian (Lucknow)	Gupta et al. [31]
0.65%	Nigerian	Ani et al. [32]
0.74%	London	Brewin et al. [30]
0.5–1%	American	Rayan [3]
1.16%	Chennai	Venkatesan et al. [33]
1.2%	American	Walden et al. [24]
1.22%	Indian	Sharma et al. [25]
1.3%	White American	Viertel et al. [22]
2.2%	Caucasian (children)	Merks et al. [13]
2.59%	Indian (cadavers)	Savgaonkar et al. [34]
2.67%	Kashmiri	Bhat et al. [35]
2.8%	African American	Viertel et al. [22]
3%	Anatolian	Gulekon et al. [36]
3.4%	Saudi Arabian	Bokhari et al. [37]
6.2%	Turkish	Erken et al. [6]
